# Is the central vein sign a useful diagnostic marker for paediatric-onset multiple sclerosis?

**DOI:** 10.1177/13524585221142318

**Published:** 2022-12-13

**Authors:** Omar Abdel-Mannan, Olga Ciccarelli

**Affiliations:** Queen Square MS Centre, UCL Queen Square Institute of Neurology, Faculty of Brain Sciences, University College London, London, UK/Department of Neurology, Great Ormond Street Hospital NHS Trust, London, UK; Queen Square MS Centre, UCL Queen Square Institute of Neurology, Faculty of Brain Sciences, University College London, London, UK/NIHR University College London Hospitals Biomedical Research Centre, London, UK

The central vein sign (CVS) is an emerging imaging biomarker that may aid in improving multiple sclerosis (MS) diagnostic accuracy.^1^ There is cumulative evidence that the proportion of CVS positive (CVS+) white matter lesions can help in differentiating MS from its mimics. In fact, a recent prospective study of 51 adult-onset MS patients demonstrated that a 40% CVS+ lesion cutoff was associated with 97% accuracy and a 96% positive/100% negative predictive value when distinguishing between MS and other diseases associated with central nervous system white matter lesions.^2^ A recent two-centre prospective study of 91 adult patients with acquired demyelinating disorders demonstrated that CVS was one of the most accurate measures differentiating MS from seropositive neuromyelitis optica spectrum disorder (AQP4 + NMOSD) (84% vs 33%, accuracy/specificity/sensitivity: 91/88/93%, *p* < 0.001).^3^ To date, few studies have looked at the diagnostic accuracy of CVS in paediatric-onset MS.

In a prospective study by Lapucci et al.,^4^ the authors aimed to compare the proportion of lesions showing a CVS between 10 paediatric-onset MS and 12 disease duration-matched adult-onset MS patients. All patients underwent 3T-MRI brain scan (Siemens MAGNETOM Prisma, Siemens Healthcare, Erlangen, Germany), with the following sequences acquired: 3D sagittal T2-FLAIR, 3D sagittal T1 MPRAGE, and 3D sagittal segmented echo-planar imaging (EPI). CVS assessment on EPI sequences was done blindly with CVS+ lesions detected through consensus by two experienced neurologists. The authors demonstrated that all adult-onset patients met the 40% threshold of CVS+ lesions, compared with 70% of paediatric-onset MS patients.

While it is important and timely to study the significance of CVS+ lesions in children with MS and how they compare to their adult counterparts, the study was limited by small numbers (an ongoing issue in most paediatric MS studies given the rarity of the condition and recruitment challenges), which resulted in an inability to show statistically significant differences between the two groups. Moreover, technical challenges need to be considered. First, the use of gadolinium may affect the cutoff: in a US study of 153 MS patients (both relapsing remitting and progressive patients), small veins in gadolinium enhancing lesions were obscured by the effects of oedema and T2 signal changes.^5^ Second, the value of the cutoff depends on T2 lesion load and technical imaging characteristics, so it may be difficult to apply it to the general MS population. Third, the scoring of the CVS requires neuro-radiological expertise,^1^ which may not be available in all centres. Finally, the application of a cutoff requires a manual check of all white matter lesions; clearly an impractical task in clinical practice when reporting on patients with high lesion loads. When Select6* and Select3* algorithms were used, they were not superior to the cutoff calculated when all lesions were considered.^3^

In conclusion, CVS remains mainly a research biomarker, which is both personnel and time-intensive and requires optimal pulse sequences that are not widely available on clinical scanners.^6^ Given that the 2017 McDonald criteria can accurately diagnose paediatric MS (with the inclusion of intrathecal oligoclonal bands and serum myelin oligodendrocyte glycoprotein, MOG, antibody testing), we would suggest that CVS is used as an additive metric for selected cases, in whom the diagnosis may be challenging, for MS confirmation, rather than part of recommended imaging protocols.^6^
